# Reimagined diabetic care approach: A qualitative study on the acceptability of mhealth interventions in a LMIC

**DOI:** 10.1371/journal.pone.0343711

**Published:** 2026-02-24

**Authors:** Ola Sukkarieh, Leonard Egede, Mona Osman, Maya Bassil, Myrna A. A. Doumit

**Affiliations:** 1 Hariri School of Nursing-American University of Beirut, Lebanon; 2 Wisconsin College of Medicine, United States of America; 3 Department of Family Medicine, American University of Beirut, Lebanon; 4 Qatar University, Qatar; 5 Hariri School of Nursing-American University of Beirut, Lebanon; Sefako Makgatho Health Sciences University, SOUTH AFRICA

## Abstract

**Background:**

Lebanon is a lower-middle income country in the MENA region that continues to be drained structurally by the socioeconomic upheaval. The estimated prevalence of T2DM in Lebanese adults is 9%. Despite the rapid growing use of mHealth and favorable health outcomes worldwide, the impact is understudied in Lebanon.

**Purpose:**

Our study aimed to assess the acceptability of the use of mHealth intervention delivered via mobile phones that promotes diabetes self-management behaviors for Lebanese patients with T2DM.

**Design and Methods:**

We used a descriptive qualitative approach for the study. Nine study participants were recruited based on purposeful and maximum variation sampling. Interviews were analyzed using the conventional content analysis.

**Results:**

Analysis of the interviews revealed four major categories: (A) Transformative Approach to Care: Feeling Safe and Secure; (B) One Approach does not fit all; (C) Addressing psychological well-being; (D) Time and Economic gains.

**Conclusion:**

This study provides compelling evidence that mHealth is highly acceptable among Lebanese adults with T2DM and offers significant potential to enhance diabetes care in LMICs. Participants embraced mHealth as a complementary tool that enhances communication, supports psychological well-being, and reduces financial barriers.

## Introduction

Type 2 diabetes mellitus (T2DM) is a rapidly expanding global health crisis, with significant morbidity and mortality [1]. The burden of T2DM is growing disproportionately in low- and middle-income countries (LMICs) compared to high-income nations, driven by rapid urbanization, lifestyle changes, and limited healthcare access [2]. According to the International Diabetes Federation (IDF) [1], the Middle East and North Africa (MENA) region has the highest regional adult prevalence—1 in 6 adults (approximately 85 million people) currently live with diabetes—and this number is projected to nearly double, reaching 163 million by 2050. Mounted evidence has shown the detrimental consequences of T2DM microvascular (such as retinopathy, neuropathy and nephropathy), or macrovascular complications (including cardiometabolic, cerebrovascular, and peripheral vascular disease) [3] that are associated with poor quality of life and adverse financial burden on households and national health care cost and economy [4].

Reducing hemoglobin A₁c (HbA₁c) by approximately 1% has been associated with significant decreases in macrovascular and microvascular complications [5], improvements in glycemic management [6], and reductions in diabetes-related distress [7]. However, sustaining optimal glycemic control for people with T2DM is perplexing and burdensome as it requires multiple daily self-management decisions and care. Embracing “no one size fits all” paradigm, as endorsed by American Diabetes Association standards of Care (2022), diabetes education self-management needs to be culturally sensitive and delivered by qualified interprofessional team to optimize self-management and glycemic and cardiometabolic outcomes (blood pressure and lipid control [8,9].

### Mobile health

With the advancement of technology, key innovative health technologies have been growing globally and exponentially, particularly in low to middle-income countries, to address health priorities and optimize health care [10]. World Health Organization (WHO) defines mHealth as “medical and public health practice supported by mobile devices, such as mobile phones, patient monitoring devices, personal digital assistants, and other wireless devices.” [10]. Mobile Health (mHealth) involves and capitalizes on the uses of mobile phones through various phones operations and applications [11]. Ample benefits on utilization of mHealth technologies for diabetes self-management reveals real-time engagement in personalized education and bi-directional communication between patients and health practitioners [8,12,13] and has shown promising glycemic [14,15] and cardiometabolic outcomes (reduction in blood pressure and cholesterol level [16,17] as well as improvement in quality of life [18]. The use of mHealth conducted in developed countries has shown similar results on uses of mHealth in developing countries [19] particularly in LMIC [20]. Several trials have demonstrated that mHealth for diabetes self-management have revealed substantial reduction in HbA1c [21–23], particularly among the highly uncontrolled participants, and better diabetes self-management [24–26].

### Context in Lebanon

Lebanon is a lower-middle income country in the MENA region that has witnessed wide range of man-made crises, namely distressing socioeconomic upheaval, COVID pandemic, Beirut blast and rapid 40% increase in population due to the influx of over 1.5 million refugees seeking asylum; which all impacted the health care system drastically and irreconcilably [27,28]. A 5-year follow-up cohort study of Lebanese patients with T2DM revealed an increase in incidence of T2DM which was 17.2 per 1000 person-years that is higher than comparable incidence studies available from the region in Greater Beirut [29] and the rate of progression to diabetes among those who had prediabetes at baseline was 18%, at 5 years [29]. In addition, diabetes care in Lebanon is suboptimal, with only 30% reported to achieve an HbA1C level below 7%, and 6% who have regular eye and foot examinations [29,30]. Indeed, the patient population with T2DM is uncharacterized mainly, and current treatment strategies remain suboptimal. However, there is compelling evidence that adhering to proper diabetes self-management can optimize clinical outcomes and quality of life, while reducing hospitalizations and healthcare costs [8].

In Lebanon, understanding the acceptability of using mHealth is imperative. A qualitative study that explored readiness to use a mobile health application in pediatric oncology patients revealed that the participants showed interest in a mobile health app for supportive pain management [31]. So far, studies have investigated the healthcare providers’ perceptions in using health records, to which they reported their willingness to use [32,33]. Such studies have been mainly restricted to use of medical records for health behavior changes. However, there are no studies that reveal that use of mHealth for delivering health education in chronic disease self-management which are quite burdensome to manage on the personal level. Therefore, the purpose of this descriptive qualitative study was to assess the acceptability of the use of mHealth intervention that promotes diabetes self-management behaviors for Lebanese patients with T2DM.

## Method

### Design

A qualitative descriptive research study design that followed the Qualitative Description (QD) which is a categorization used in descriptive qualitative research, mainly for exploring health care and nursing-related phenomena [34]. This approach is particularly suitable when the purpose of a study is to obtain straightforward, rich, and direct descriptions of participants’ experiences, perceptions, or views. Unlike other qualitative methodologies QD does not aim to generate theory or interpret deep meanings, but rather to capture how people understand and describe a phenomenon in their own words**.** Hence we aimed at assessing acceptability of a new approach to diabetes education in Lebanese adults with T2DM

### Participants

Nine study participants were recruited based on purposeful sampling, saturation principles, and according to the following criteria: Lebanese adult;2) age 18 years or above; 3) able to communicate in Arabic; 4) has a clinical diagnosis of T2DM; 5) willingness to share their ideas about mHealth. Saturation was reached with the seventh participant, as no new ideas emerged from the participants, and the data began to repeat [35–36]. We opted to add two additional participants to ensure that no new ideas emerged. The most useful sampling for the naturalistic approach is maximum variation sampling, which was followed in this study [37]. Variation was based on age, gender, education, socio-cultural, and religious background. Participants were excluded if they had active mental illness upon recruitment, confusion or were insulin-dependent It is worth noting that insulin-dependent individuals were excluded, as their glycemic control is primarily driven by insulin therapy. Consequently, the role and design of mHealth interventions for this population differ from those for non–insulin-dependent individuals, where behavioral modifications more directly influence outcomes.

### Recruitment of participants

Participants were recruited from two clinical sites in major medical center in Beirut (Private and Public). Following the approval of the Institutional Review Board (IRB), the physicians of all sites initially sought participants’ approval to be contacted by the primary investigator of the study. Following the participants’ consent to be contacted, the PI contacted candidate participants to confirm their agreement to participate in the study and to arrange a convenient appointment. Co-PI secured the participants’ written consent through their signatures before the interview. All face-to-face interviews were conducted in a quiet environment to ensure privacy and smooth interviewing process.

#### Sample description.

The sample consisted of six females and three males; age ranged between 52 years and 77 years with a mean age of 62 years. Participants experience with T2DM ranged between five and eleven years (Table 1.)

**Table 1:** Demographic characteristics of participants (N = 9).

Variable	Category	n
Gender	Female	6
	Male	3
Age (years)	Mean	62
	Range	52–77
Marital status	Married	7
	Single	1
	Widowed	1
	Divorced/Separated	0
Educational attainment	University	5
	High school	1
	Middle school	0
	Elementary	3
	Illiterate	0
Employment status	Housewife	4
	Retired	2
	Employed	3
Financial status	Able to make ends meet	5
	Unable to make ends meet	3
	Comfortable/more than enough	1
Health insurance	Yes	6
	No	3
Duration of diabetes	≥11 years	7
	5–10 years	2
Received diabetes information from a health care provider	Yes	6
	No	3

#### Setting.

All interviews took place in a private room at a convenient place for the participant.

### Interviews

In-depth interviews and field notes were used as data sources to understand the participants ‘acceptability regarding the use of a mobile phone as a tool for providing them with information, teaching material, and follow-up about their diabetic condition. At the beginning of each interview, the corresponding Co-PI who is a seasoned qualitative researcher re-explained the purpose of the study and provided the participant with an idea about communicating with him/ her through a phone to deal with his/ her diabetes. Data were collected between April 2025 and June 2025(02/04/2025− 19/06/2025). Interviews were conducted in Arabic (the native language of the participants) by the corresponding Co-PI. In each interview, the participant was the major speaker, and the Co-PI was mainly a listener and a facilitator. The participants were reminded that their participation was voluntary and that at any time they could decline or withdraw from the study without any consequences.

The interviews were audio-taped, and field notes were recorded. Each interview was coded so that only the researcher knew the identity of the participants. Participants were assured of confidentiality, and pseudonyms were used. The code list and the original tapes are kept in a locked file cabinet in the PI’s office for three years, at which time the notes will be destroyed and the recordings erased. Each interview took around 40–50 minutes. Interviews were transcribed by a research assistant and transcriptions were checked verbatim by the co-PI. Interviews were translated and back translated by two different persons who are bilingual, and both versions were compared to ensure that the conceptual equivalence was maintained. The translators and transcriptionist signed a confidentiality form

The interview was based on the three major domains revolving around accessibility, application, concerns, and design, respectively: 1) Is mHealth tech feasible/accessible by the target population? 2) What would patients want to use mHealth for? What would they not want to use it for? 3) What are important facilitators and barriers with regards to the design and delivery of mHealth technology?

Probing was tailored to the participant’s narration, such as: “Please tell me more about it; can you elaborate more on this point? please give me an example. To prevent socially pleasing answers, the Co-PI clarified at the beginning of the interviews that she was concerned about the participants’ ideas, views, and concerns about the use of mHealth.

### Data analysis

Interviews were analyzed using conventional content analysis (CCA), as recommended by Hsieh and Shannon (2005). Conventional content analysis is generally used in a study design that aims to describe a phenomenon, in this case, the acceptability of using a mobile application. This type of design is usually appropriate when existing theory or research literature on a phenomenon is limited, as is the case in this study. In conventional content analysis, researchers avoid using preconceived categories [38], instead allowing the categories and their names to emerge from the data, and they immerse themselves in the data to allow new insights to emerge [38].

Data analysis was done following the guidelines as recommended by Hsieh and Shannon [38]. The PI and Co-PI, each individually, read all the data repeatedly to achieve immersion and gain a sense of the whole. Then, the data was read word by word to derive patients’ codes, first highlighting the exact words from the text that appeared to capture key thoughts or concepts. Next, the PI and Co-PI approached the text by taking notes of their initial impressions, thoughts, and analysis. As this process continued, labels for codes emerged that reflected more than one key thought. Codes were then sorted into categories based on how they are related and linked. These emergent categories were used to organize and group codes into meaningful clusters, then exemplars for each code and category were identified from the data. The PI and Co-PI, who are bilingual (English, Arabic), ensured to maintain the conceptual equivalence of what participants verbalized during interviews by reviewing the translation and back translation done by translators [39]. Data collection and analysis were done concurrently throughout the study. It is worth noting that the level of concurrence in data analysis between the PI and Co-PI was about 95% and in case of disagreement, they reverted to the text for proper judgment. It warrants mention that all authors contributed to the conceptualization of the study, as well as to the discussion and write-up.

### Ethical considerations

The study received Institutional Review Board (IRB) approval from the affiliated institution of the PI (American University of Beirut- SBS-2024–0285). At the beginning of the interview, each participant was invited to read and provide a written a consent form. The forms were prepared in Arabic and English language and each participant was given the choice between the two versions. All participants chose the Arabic version.

### Rigor

In keeping with the naturalistic approach, to ensure credibility, within-method triangulation for data collection, peer debriefing, and member check were followed. Lincoln and Guba [37] believe that a study meets the criterion of transferability when findings can be applied in different contexts. In this study, the Co-PI interviewed participants who varied in terms of age, educational level, marital status, years since diagnosis, and current activities, which contributed to validating the themes identified in the interview data. Dependability and confirmability were ensured through intercoder reliability, as suggested by Polit and Beck [34], and by selecting and integrating participants’ quotes to describe the results, which contributes to the neutrality of the research findings.

## Findings

Four themes that illuminate the participants’ viewpoints on this new approach for diabetic care were identified. It is worth noting that these themes represent the first substantiated approach to using an mHealth for follow-up on diabetic care in Lebanon. All participants stated clearly that mHealth will not replace physicians’ visits, but it could be used as a complementary approach for follow-up. Moreover, it is worth noting that none of the participants expressed skepticism towards using mHealth.

1) Transformative approach to care: feeling safe and secure

All participants expressed a feeling of safety and security when providing feedback about the use of mHealth. They described the mHealth as a new approach to care that would give them a sense of security and safety. They all mentioned that this new approach will make them feel supported by the healthcare team and that they will not be left on their own, which gives them a sense of reassurance.

Dani, a 77-year-old participant with 11 years of diabetes, said: “I like the feeling that my health team is interested in me and they are following up with me…. This shows that we still have morals… if there is no politics in it, I am very ok…”

Jimmy a 65 years old participant with also 11 years of diabetes also mentioned,” For me, this approach is perfect and I feel contented and happy because a health team will follow me up….. I feel safe and I am well followed up…..I feel secure

Indeed, feeling secure was closely tied to feeling surrounded. The participants explained that mHealth would make them feel heard and attended to their needs in a timely fashion. As such, they would not feel left out in managing their diabetes regime alone.

Biggs a 52 years participant with more than 11 years of diabetes said:”…. It feels fantastic to know that professional people are ready to listen to you according to your needs. This feeling empowers me as I think that professionals surround me…. This technology will help me to feel connected wherever I travel ….”

Selwa a 57 years participant with 11 years with diabetes mentioned “ … this approach is excellent and it will help for easy follow-up. I always have questions, but I do not call my physician every time because I do not want to bother him, but now with this approach, I can pose all my questions…”.

2) One Approach does not fit all

Two aspects surfaced under this theme: usability and individualization. Regarding usability, all participants welcomed the use of mHealth, particularly short video calls. They perceived it as an easy and practical tool to expand their knowledge on diabetes self-management. However, all participants’ views consolidated around individualization of use in terms of content and delivery process. In this regard, each participant had a different concern and request regarding language, time of call, and materials to be shared. Some prefer to be called in the morning; others in the afternoon, and some in the evening. They also stressed on receiving relevant information that addresses to their diabetes regime rather than broad knowledge.

Batoul a 52 years old participant with 7 years with diabetes said “I do not mind if I share with a group my concerns…. I wish to have information about the appropriate food that I need to take … I like to listen to others’ concerns because I would learn from them….”

However, Alida a 67 years old participant with 12 years of diabetes emphasized privacy “….I do not like to share the call with other patients. I do not want anyone to know about my condition and the type of questions I ask. Privacy is important to me; information needs to be tailored to my needs and not general. I prefer individualized information between the health team and me only….”.

3) Addressing psychological well-being

Participants mentioned the negative psychological toll of being a patient with diabetes. They all hoped that this mHealth approach would also tackle this aspect of care that they think is neglected. In addition, participants verbalized the fear messages that they receive from health care practitioners and emphasized the need for positive tone and framing of the delivered information on diabetes self-management.

Gigi a 69 years old participant with 13 years of diabetes said:“….there should be among the team psychologists because diabetes has an impact on the psychology … the psychologist plays an important role. It is neglected in the care….”

Maria a 52 years old participant with 6 years of diabetes said “…. the psychological support is essential for the patient with diabetes. The way we communicate with the patient is meaningful. Positive communication is a must….”

“In psychological follow-up, you don’t want someone to call and talk with you and listen—you just want things like phrases and quotes… they help a bit.”

4) Time & financial gains:

All participants verbalized the convenience of the utilization of mHealth, which saves mainly time and money spent on resources and physicians’ visitation fees. Some mentioned that their care does not get interrupted during travel. They also acknowledged that mHealth fits the purpose of technology in terms of convenient outreach. The participants highlighted the current economic crisis and the affordability of medical care and perceived that mHealth can help save costs.

Dani a 77 years old participant with 14 years of diabetes said:”… it saves the cost of commuting, traffic, and energy … and it will decrease the need for visiting the doctor….”

Jimmy a 65 years ola participant with 13 years of diabetes said:”… I do not mind paying minimal fees for the service, it would still be cheaper than visiting the physician, particularly in our current economic situation…”

It is worth noting that despite highlighting that mHealth would decrease their care cost, all participants mentioned that mHealth will not replace the physician’s visits but might decrease the frequency.

## Discussion

In this study, we investigated the acceptability of using mHealth as a supportive tool for diabetes self-management among Lebanese adults with Type 2 Diabetes Mellitus (T2DM). The findings demonstrate a positive reception to mHealth among participants, with emphasis on its ability to offer psychosocial support, enhance disease self-management, and address logistical barriers such as cost and accessibility. This approach aligns with the results of a study by Drake af Hagelsrum and colleagues [40], which highlights the importance of understanding users’ perceptions of mHealth services when developing person-centered digital interventions. These findings are significant in the Lebanese context, where ongoing economic instability and strain on the healthcare system call for innovative, scalable, and patient-centered solutions.

Participants strongly associated mHealth with a sense of safety, security, and continuous support with healthcare providers. This sense of being “connected and not alone” aligns with previous research, which shows that mHealth can enhance patients’ perception of being monitored and cared for, thereby improving treatment adherence, sense of control, and empowerment in decision-making [13,41]. Consistent with previous literature [40], mHealth was viewed as a means to bridge the continuity of care gap between clinic visits which is a critical element in chronic disease care. As chronic diseases mandate daily management, having the appealing feature of mHealth to serve as a “constant companion”—especially during stressful times like travel, crisis, or health deterioration—was valued by participants. The findings compare to a study conducted by McCurdie and colleagues [42] on the role of mHealth as a “care companion” that bridges gaps in time, geography, and emotional access. Similarly, Lyles and colleagues [43] found that such tools contribute to a sense of “being watched over,” especially in low-income settings where routine in-person follow-up is often disrupted.

The use of mHealth is further valued due to its cultural congruence. In the MENA context, where collectivism and interpersonal connectedness are core values, the concept of “being followed up” resonates deeply. One participant in the current study noted that “this shows that we still have morals”—a powerful expression of how health interventions are judged not only by their utility, but also by their perceived humanity and ethical alignment. For Lebanese participants in this study, this sense of being monitored by a competent care team was particularly valued given their fragmented and under-resourced healthcare status quo [27,28].

Participants consistently emphasized the need for individualized and culturally sensitive content. While all found mHealth usable, preferences varied widely regarding the mode (e.g., video vs. text), timing (morning vs. evening), group vs. individual interaction, and content focus (diet, exercise, emotional support). Lauffenburger et al. [44] and Hagelsrum and colleagues [40], in two qualitative studies conducted in the US and Sweden, respectively, emphasized the importance of tailoring mHealth interventions according to individuals’ needs and requests. The results of this study highlight the importance of treating every individual as a unique being with specific needs and requests. Indeed, a systematic review of technology-enabled Diabetes Self-Management Education, conducted by Greenwood and colleagues [13], reinforced those interventions were most effective when they included individualized feedback, tailored education, and bidirectional communication—three features that enhance not only glycemic outcomes but also psychosocial well-being and user satisfaction. Our study contributes to this evidence by revealing that, in an LMIC context such as Lebanon, personalization also addresses privacy concerns, language nuances, and time constraints.

The psychological impact of T2DM was highlighted as a neglected yet critical component of care. Participants called for the inclusion of psychological well-being support within mHealth content, particularly emphasizing the need for positive communication, unlike the conventional fear-based communication. The mHealth content and interactions should convey positive messages to patients and encourage them to approach their diabetes more positively [45]. Sending positive quotes every morning was verbalized as one strategy that alleviates the psychological burden. In comparison, the findings from Lauffenburger and colleagues [44] emphasized the importance of positive communication as a means of contacting patients. This approach is considered a pivotal point in the process of mHealth adoption and acceptance. The use of uplifting messages and access to professional psychological support through mobile platforms could foster a more holistic and humane approach to diabetes care. Incorporating psychological well-being and healthy coping as mandated by the American Diabetes Association [46] standards of care, even if using a mHealth venue, is foundational for achieving positive health behaviors. For populations facing political instability and socioeconomic hardship, such as in Lebanon, this dual focus on physical and psychological well-being is particularly essential to mitigate the burden of diabetes self-management.

Participants acknowledged the financial and logistical burdens associated with frequent clinic visits, especially amid Lebanon’s economic collapse. mHealth was perceived as a time-saving and cost-effective alternative that allows continuity of care regardless of geographic or financial limitations. This is consistent with Hearn and colleagues [20] and Kruse and colleagues (2019) [19], who found that mHealth interventions were not only clinically effective but also financially advantageous in LMICs. Participants in this study were also open to paying a small fee for mHealth services, viewing them as more affordable than conventional physician visits. This insight highlights the potential sustainability of mHealth models that blend minimal patient fees with continuous medical support.

In the Lebanese context, where the World Bank [27] has documented a dramatic increase in poverty, out-of-pocket health spending, and a mass migration of healthcare professional and Lebanese citizens, participants’ feedback reflects mHealth’s potential to offset systemic inefficiencies and address equity gaps. For instance, some participants in the study noted that mobile follow-up would enable them to continue their care regimen while traveling or during times of political unrest, thereby further enhancing the resilience and continuity of their treatment. Furthermore, participants viewed the ability of mHealth to reduce indirect costs, such as emotional strain, transportation, and waiting time. These savings have been shown to be especially valuable to patients with chronic diseases, who require lifelong monitoring and regular counseling that would eventually improve self-efficacy, adherence, and reduced hospitalizations, emergency visits, and complications [47]. Notably, this theme illustrates that mHealth could serve as a resilience mechanism for strained health systems by reducing clinical load and offering remote support. As Lebanon continues to face healthcare workforce shortages and resource constraints, integrating mHealth may offer a scalable, efficient adjunct to traditional care models. In view of the stability feelings that the idea of mhealth provides to patients with diabetes in Lebanon, it would be beneficial to integrate this approach in the care provided by the public and private sector mainly in Primary Health care centers that provide care to the less privileged population.

A key strength of this study lies in its qualitative descriptive design, which allowed in-depth exploration of patients’ nuanced perceptions, rooted in personal experience. The use of maximum variation sampling ensured diversity across sociodemographic and clinical characteristics, increasing the transferability of findings which is an important goal of this type of qualitative study.

Further, this study assessed acceptability rather than actual use. Future research should include pilot testing and evaluating real-world usability and outcomes.

## Conclusion

This study provides compelling evidence that mHealth is highly acceptable among Lebanese adults with T2DM and potentially other non-communicable diseases as well and offers significant potential to enhance diabetes care in LMICs. Participants embraced mHealth as a complementary tool that enhances communication, supports psychological well-being, and reduces financial barriers. mHealth represents not only a technological innovation, but also a person-centered approach to diabetes care, especially in settings where healthcare infrastructure is under significant strain. **Notably, the study highlights that culturally responsive, contextually grounded mHealth interventions can be effectively integrated even in resource-constrained environments. The study recommends a scalable, adaptable framework that can be replicated and adapted to diverse cultural and healthcare contexts beyond Lebanon. Moreover, it adds to the limited yet growing body of evidence from the Middle East and similar LMICs, offering insights that can inform global efforts to reduce diabetes-related disparities.** Importantly, in LMICs where patients may differ widely in digital literacy, education level, and access to technology, personalization also acts as an equity-enhancing strategy. mHealth platforms, when used by health professionals and persons with diabetes as described, could accommodate rather than exacerbate disparities, providing tailored support to those who may otherwise be marginalized in a conventional medical model.
